# Identification and Evaluation of Novel Drug Targets against the Human Fungal Pathogen *Aspergillus fumigatus* with Elaboration on the Possible Role of RNA-Binding Protein

**DOI:** 10.18869/acadpub.ibj.21.2.84

**Published:** 2017-03

**Authors:** Saeid Malekzadeh, Soroush Sardari, Parisa Azerang, Dorsa Khorasanizadeh, Solmaz Agha Amiri, Mohammad Azizi, Nazanin Mohajerani, Vahid Khalaj

**Affiliations:** 1 Fungal Biotechnology Unit, Medical Biotechnology Department, Biotechnology Research Center, Pasteur Institute of Iran, Tehran 1316943551, Iran; 2 Bioinformatics and Drug Design Unit, Medical Biotechnology Department, Biotechnology Research Center, Pasteur Institute of Iran, Tehran 1316943551, Iran

**Keywords:** *Aspergillus fumigatus*, RNA-binding protein, Peptidylprolyl isomerase, Juglone, RNA interference

## Abstract

**Bakground:**

*Aspergillus fumigatus* is an airborne opportunistic fungal pathogen that can cause fatal infections in immunocompromised patients. Although the current anti-fungal therapies are relatively efficient, some issues such as drug toxicity, drug interactions, and the emergence of drug-resistant fungi have promoted the intense research toward finding the novel drug targets.

**Methods:**

In search of new antifungal drug targets, we have used a bioinformatics approach to identify novel drug targets. We compared the whole proteome of this organism with yeast *Saccharomyces cerevisiae* to come up with 153 specific proteins. Further screening of these proteins revealed 50 potential molecular targets in *A. fumigatus*. Amongst them, RNA-binding protein (RBP) was selected for further examination. The aspergillus fumigatus RBP (AfuRBP), as a peptidylprolyl isomerase, was evaluated by homology modeling and bioinformatics tools. RBP-deficient mutant strains of *A. fumigatus* were generated and characterized. Furthermore, the susceptibility of these strains to known peptidylprolyl isomerase inhibitors was assessed.

**Results:**

*AfuRBP*-deficient mutants demonstrated a normal growth phenotype. MIC assay results using inhibitors of peptidylprolyl isomerase confirmed a higher sensitivity of these mutants compared to the wild type.

**Conclusion:**

Our bioinformatics approach revealed a number of fungal-specific proteins that may be considered as new targets for drug discovery purposes. Peptidylprolyl isomerase, as a possible drug target, was evaluated against two potential inhibitors, and the promising results were investigated mechanistically. Future studies would confirm the impact of such target on the antifungal discovery investigations

## INTRODUCTION

*A* *spergillus fumigatus* is currently the most important airborne fungal pathogen and the leading cause of invasive aspergillosis (~85% of cases)[1]. Despite the introduction of new effective antifungal drugs, the mortality rate of invasive aspergillosis still exceeds 40%[2]. Furthermore, the emergence of drug resistance adds more complications to the treatment process. Hence, new antifungal agents are urgently needed.

While the classical cell-based screening method has been successfully used in drug discovery process[3], it is an expensive, time-consuming and labor-intensive approach[4]. Recent advances in application of computation methods in genome, proteome, and metabolome mining coupled with complete genome sequences of pathogen organisms have opened an alternative path toward identifying new drug targets. For instance, several reports have confirmed the usefulness of comparative genome analysis in identification of pathogen-specific drug and vaccine candidates[5-7].

The genome of *A. fumigatus* contains ~9900 genes, but few antifungal targets have been developed yet[8]. In the present study, we compared the whole proteome of *A. fumigatus* with *Saccharomyces cerevisiae*, as a non-pathogen fungal organism, to identify *Aspergillus*-specific proteins. Amongst the proteins, those with close homologues to human were removed, and the final targets were selected. One novel target, the aspergillus fumigatus RNA-binding protein (AfuRBP), was selected and found to be a peptidylprolyl isomerase (PPI). For further functional studies, *AfuRbp* knock out and knock down mutant strains were generated. MIC assay with known inhibitors, phenylglyoxal and juglone, and a synthetic derivative of juglone confirmed the higher sensitivity of the mutants to these compounds when compared to the wild-type strain.

## MATERIALS AND METHODS

### Comparative analysis of *A. fumigatus* and *S. cerevisiae* proteome

The identification of novel drug targets against *A. fumigatus*, as a human pathogen, was based on alignment of its proteome with the proteome of *S. cerevisiae*. The proteomes of these two fungi were extracted from NCBI (https://www.ncbi.nlm.nih.gov/genome/browse/). Three different approaches were used to identify and confirm those sequences that are present in *A. fumigatus* but are absent or significantly diverge from *S. cerevisiae*. LAST (http://last.cbrc.jp)[9] has been used as a fast sequence alignment tool to identify unique sequences for *A. fumigatus*. LAST uses variable-length (spaced) seeds realized by a suffix array[10]. The expected value of 10 (-e84) was chosen to obtain higher sensitivity (-m1000000), and the other settings and parameters were used as default. In the next step, unique sequences from *A. fumigatus* were compared using suffix tree analysis by Mummer (version 3.0) (http://mummer.sourceforge. net)[11]. Minimum maximal match was seven to restrict output proteins, but other settings were chosen as default. Finally, the proteins that were unique to fungi were identified by BlastP (NCBI Blast v. 2.2.1)[12]. Modeling was carried out by I-TASSER server (Michigan University, USA)[13-15]. Docking of the compounds was performed by HEX 6.3 software when required.

### Strains, plasmids, and culture conditions

*A. fumigatus* akuAKU80 and its *pyrG^-^* derivative (akuAKU80 pyrG-) with a highly efficient homologous recombination background were used for isolation of *AfuRbp* gene homologue and gene disruption or gene silencing experiments. *E. coli* Top10 (Invitrogen, USA) cells and pGEM–T Easy cloning system (Promega, USA) were used in all DNA recombinant procedures. Plasmid PGEM-GlaA comprising *Aspergillus niger* glucoamylase A (glaA) promoter and glaA termination signal was used for preparation of RNAi plasmid. All molecular methods including PCR and RT-PCR were performed based on established protocols[16]. In RT-PCR settings, 1 µg of total RNA was used in cDNA synthesis reactions and the *A. fumigatus* actin gene (AFUA_6G04740) was used as a loading control. Fungal strains were grown and kept on SAB agar or SAB agar medium supplemented with uridine and uracil. Modified Vogel’s medium[17] was used in isolation of fungal transformants.

### Construction of *AfuRbp* deletion/silencing cassettes and strains

**Fig. 1:**
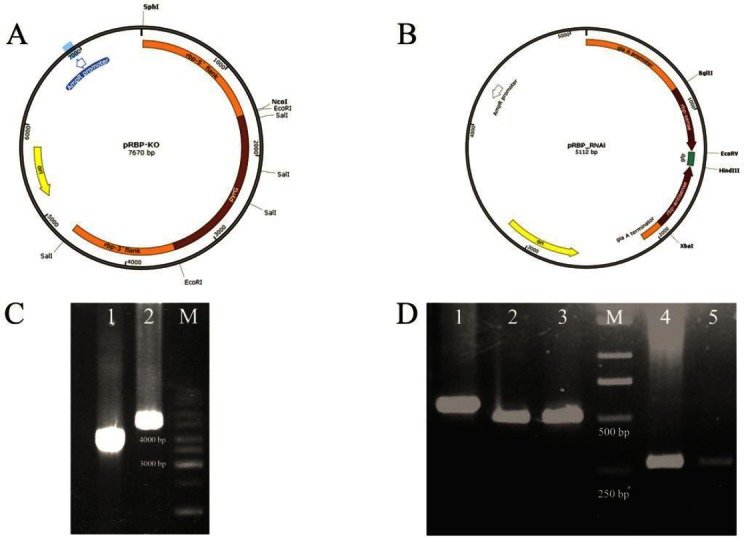
Gene constructs and molecular validations. Schematic representation of constructed plasmids used in gene deletion (pRBP-KO) (A) and RNAi (pRBP_RNAi) (B) experiments. (C) PCR analysis of wild-type (lane 1) and deletant strain (lane 2) genome using primers RBP-F1 and RBP-R1. The amplification of a 4.6-kb product confirmed the replacement of native *AfuRbp* with the disrupted fragment. (D) RT-PCR analysis of RNAi transformant grown in maltodextrin or glucose medium for 24 h. Lane 1, actin fragment amplified from the genome (560 bp); lane 2, actin fragment amplified from cDNA originated from glucose; lane 3, maltodextrin cultures (485 bp); lane 4, AfuRbp expression level in RNAi transformant grown in glucose medium; lane 5, maltodextrin medium. *AfuRBP*-specific RT-PCR primers, RBP_rt1/rt2, amplified a 300-bp product. M, DNA size marker

For construction of the *AfuRbp* deletion cassette, a sequential cloning strategy was applied as described before[18]. Briefly, 1.5 kb of 5’ and 1.2 kb of 3’ flanking regions of the gene were amplified separately, using RBP_KO1/RBP_KO2 (containing *Sph*I/*Nco*I restriction sites) and RBP_KO3/RBP_KO4 (containing *Eco*RI/*Sal*I restriction sites) primer sets, respectively (Table 1). These fragments were cloned next together in pGEM-Teasy vector. At the final step, the *A. fumigatus* *pyrG* gene, as a selection marker, with its own promoter and terminator containing *Eco*RI site at both ends was cloned into *Eco*RI site, between the 5’and 3’ flanking regions, resulted in pRBP-KO (Fig. 1A).

**Table 1:** Primers used in this study

Name	Sequence (5`-3`)	Enzymes
RBP_KO1	GGGCCC*GCATGC*TCTCTTCAGCTCTGTGGTTG	*Sph*I
RBP_KO2	*CCATGG*CATACACTCGAGCACGGTCG	*Nco*I
RBP_KO3	*GAATTC*AATGCGTGATGTTG	*Eco*RI
RBP_KO4	*GTCGAC*GTCAGTTGACAGAGCGAG	*Sal*I
RBP_SENSE_F	G*AGATCT*CCATCCCATTGTTGTT	*Bgl*II
RBP_SENSE_R	T*GATATC*TGCTCCTGCTGTCGGCGT	*Eco*RV
RBP-F	AAGTTCATGGAAAAGTGGGC	*-------*
RBP-R	AGTCAGTTGACAGAGCGAGG	*-------*
RBP_rt1	CCCCTGAGGTTGAGGAGCGG	*-------*
RBP_rt2	CGAGGTTCTGCACTTTGGCG	*-------*
GFP_F	*GATATC*GTCCAGGAGCGCAC	*Eco*RV
GFP_R	*AAGCTT*CTCGATGCGGTT	*Hind*III
ACT-F	ATGTCACTGTGCAGATTGTC	*-------*
ACT-R	CGTAGAGGGAGAGAACGGCC	*-------*

To generate the *AfuRbp* silencing cassette, an inducible RNAi transcriptional unit was designed based on a previously described protocol[19, 20]. This unit was driven by the glaA promoter and comprises an approximately 500 bp Rbp fragment (sense, nucleotides 342-841), followed by a 100-bp buffer EGFp fragment and the same ~500 bp Rbp fragment in the opposite direction (antisense). Sense and antisense fragments were amplified by PCR using primers RBP_SENSE_F and RBP_SENSE_R carrying appropriate restriction sites (Table 1) and cloned into PGEM-GlaA vector. A 100-bp EGFP fragment was then amplified by PCR using specific primers (Table 1) and cloned between the latter fragments to generate final silencing cassette (pRBP_RNAi) (Fig. 1B).

The prepared constructs were used to transform *A*. *fumigatus* KU80ΔpyrG strain as described before[21]. Positive transformants were selected on Vogel’s minimal medium lacking uracil/uridine supplements. To find the knock out strains, transformants were screened by PCR using primers RBP-F and RBP-R (Table 1). As the silencing construct, pRBP_RNAi did not contain any fungal selection marker, the pRG3- AMA1 vector containing the pyrG selection marker was used as the second plasmid in co-transformation reaction. RNAi transformants were also selected based on PCR using *gfp*- and *AfuRbp-*specific primers.

### Susceptibility testing

**Fig. 2:**
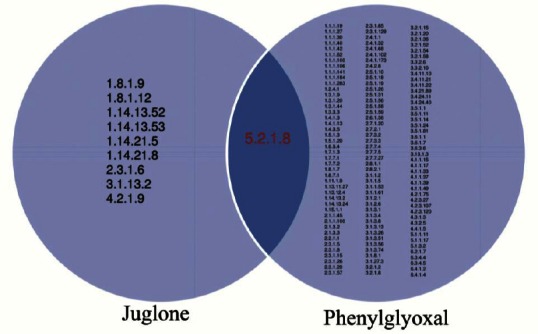
List of enzymes (EC number) targeted by juglone or phenylglyoxal. The peptidylprolyl isomerase is the only overlap target for both juglone and phenylglyoxal as inhibitors.

MICs were determined based on CLSI Broth Microdilution Method (M38-A2 Document) with some modifications. Briefly, fungal spores (10^4^/well) were inoculated in 96-well microtiter plates containing RPMI 1640 medium enriched by 2% glucose (or maltodextrin), and the susceptibility was assessed at a final compound concentration range of 0-200 µg/ml after 48 h. All MIC assays were performed in triplicates. Based on orthology and EC number (EC: 5.2.1.8) of AFUA_2G07650 (AfuRBP), two organic inhibitors of the PPI activity, i.e. phenylglyoxal and juglone (5-hydroxy-1,4-naphthoquinone), were found by searching BRENDA enzyme database (www.brenda-enzymes.info) and tested (Fig. 2). A synthetic derivative of juglone, 5-O-Acetoxy-1,4-naphthoquinone (juglone acetate, JA), was also prepared using a previously published method[13] and assessed in MIC tests. This derivative was made to make the compound more lipophilic.

### Docking and homology modeling

For bioinformatics assessment of the chemical inhibitors used in MIC assays, the 3D structure of AfuRBP was predicted by I-TASSER. The predicted binding sites were subsequently used for docking of the inhibitors. HEX 6.3, ArgusLab (v.4.0.1, http://www.arguslab.com/arguslab.com/Publications.html), and WebLab Viewer Lite (v. 4.2) were used for docking of the inhibitors and manipulation of the protein.

## RESULTS

### Identification of target proteins based on *in silico* comparative analyses

Based on the LAST alignment of the *A. fumigatus* proteome (9630 proteins) against *S. cerevisiae*, 474 unique proteins were recognized in *A. fumigatus* as the homologous of these proteins were not present in *S. cerevisiae*. All of the 474 proteins were further examined in KEGG, NCBI, EXPASY, and EBI databases. It was found that the functions of 161 proteins have already been explained, and their accession numbers are available. The remaining 313 proteins were found to be hypothetical (data not shown). Finally, 50 out of 161 protein candidates were chosen as they were fungal-specific but not present in *S. cerevisiae* (Table 2). The RBP, as a potential drug target, was selected for further evaluation. This protein is encoded by an 1158-bp gene located on chromosome 2 and has been annotated as a PPI.

**Table 2:** List of selected proteins specific to fungi other than *S. cerevisiae*

No.	Accession no.	Description	Taxonomy	Orthology
1	AFUA_8G06570	Acetylxylan esterase ((EC:3.-.-.-)	Dikarya	Acetylxylan esterase [EC:3.1.1.72]
2	AFUA_4G00860	Cell surface protein	Pezizomycotina	
3	AFUA_1G13450	Cell wall proline-rich protein	Pezizomycotina	
4	AFUA_6G14090	CFEM domain protein	Leotiomyceta	
5	AFUA_4G12450	Conserved lysine-rich protein	Pezizomycotina	
6	AFUA_4G13230	Developmental regulatory protein WetA	Saccharomyceta^†^	
7	AFUA_8G00250	Dimethylallyl tryptophan synthase (EC:2.5.1.-)	Leotiomyceta	N-dimethylallyltransferase [EC:2.5.1.-]
8	AFUA_7G01280	DNA damage response protein RcaA	Metazoa	
9	AFUA_7G06740	Endoglucanase (EC:3.2.1.4)	Metazoa	Endoglucanase [EC:3.2.1.4]
10	AFUA_6G11980	Exo-beta-1,3-glucanase (EC:3.2.1.58)	Dikarya	glucan 1,3-beta-glucosidase [EC:3.2.1.58]
11	AFUA_6G14070	Extracellular glycine/serine-rich protein	*Neosartorya fischeri* & *Aspergillus fumigatus*	
12	AFUA_5G01620	Extracellular proline-rich protein	Eurotiomycetidae	
13	AFUA_8G07090	Extracellular proline-serine-rich protein	Dikarya	
14	AFUA_7G02060	Extracellular serine-rich protein	Leotiomyceta	
15	AFUA_7G02460	Extracellular serine-rich protein	Trichocomaceae	
16	AFUA_6G00670	Extracellular serine-rich protein	Dikarya	
17	AFUA_7G00320	Extracelular proline-glycine-rich protein	Leotiomyceta	
18	AFUA_7G05650	Glutamine-serine-rich protein MS8	Leotiomyceta	
19	AFUA_7G04870	Glutamine-serine-proline-rich protein	Saccharomyceta^†^	
20	AFUA_6G10580	GPI anchored CFEM domain protein	Dikarya	
21	AFUA_8G04860	GPI anchored glycoprotein	Leotiomyceta	
22	AFUA_6G14010	GPI anchored protein	Leotiomyceta	
23	AFUA_4G09600	GPI anchored protein	Metazoa	
24	AFUA_7G00970	GPI anchored serine-threonine-rich protein	Leotiomyceta	
25	AFUA_4G08295	Histone h1.3.	Leotiomyceta	
26	AFUA_8G07060	Hydrophobin	Leotiomyceta	
27	AFUA_8G01790	Integral membrane protein	Leotiomyceta	
28	AFUA_6G04280	Integral membrane protein	Leotiomyceta	
29	AFUA_5G00620	Integral membrane protein	Leotiomyceta	
30	AFUA_1G11380	Integral membrane protein	Leotiomyceta	
31	AFUA_8G01190	Isoamyl alcohol oxidase	Leotiomyceta	
32	AFUA_5G03980	LysM domain protein	Leotiomyceta	
33	AFUA_6G03050	Oleate delta-12 desaturase (EC:1.14.99.-)	Dikarya	omega-6 fatty acid desaturase (delta-12 desaturase)
34	AFUA_8G02230	Serine-leucine-rich repeat protein	*Aspergillus fumigatus*	
35	AFUA_1G13830	Threonine-rich protein	Eurotiomycetidae	
36	AFUA_1G02270	ARS-binding protein Abp2	Ascomycota	
37	AFUA_5G05990	AT DNA-binding protein	Leotiomyceta	
38	AFUA_3G08110	Cell wall protein	Ascomycota	
39	AFUA_5G09580	Conidial hydrophobin Hyp1/RodA	Leotiomyceta	
40	AFUA_5G09010	Conserved glutamic acid-rich protein	Pezizomycotina	
41	AFUA_3G12790	Conserved glutamic acid-rich protein	Eurotiomycetidae	
42	AFUA_1G05290	Endo-1,3(4)-beta-glucanase (EC:3.2.1.6); K01180 endo-1,3(4)-beta-glucanase [EC:3.2.1.6]	Fungi	endo-1,3(4)-beta-glucanase [EC:3.2.1.6]
43	AFUA_3G13110	Extracellular serine-threonine-rich protein	Leotiomyceta	
44	AFUA_5G14210	Glucose repressible protein Grg1	Dikarya	
45	AFUA_1G11220	GPI anchored protein	Trichocomaceae	
46	AFUA_1G03630	GPI anchored serine-threonine-rich protein (EC:3.1.1.5) lysophospholipase	Fungi	
47	AFUA_5G13725	Integral membrane protein	Pezizomycotina	
48	AFUA_3G08670	Integral membrane protein	Leotiomyceta	
49	AFUA_3G00830	Pfs domain protein	Leotiomyceta	
50	AFUA_2G07650	RNA-binding protein (EC:5.2.1.8); K01802 Peptidylprolyl isomerase [EC:5.2.1.8]	Trichocomaceae	peptidylprolyl isomerase [EC:5.2.1.8]

Orthology is specified where data exist.

† excluding *S. cerevisiae*

### Disruption of AfuRbp in *A. fumigatus*

The *Sph*I/*Sal*I pRBP-KO fragment was used for transformation of *A. fumigatus* (akuAKU80 pyrG-) protoplasts. Through the PCR screening of transformants, a deletion strain was isolated and examined further. In this transformant, RBP-F1 and RBP-R1 primers amplified a fragment of ~4.6 kb (KO) instead of a 3.8-kb wild-type fragment, confirming the replacement of native gene with disrupted one (Fig. 1C). RT-PCR analysis of the deletant revealed that *AfuRbp* does not express in this mutants (data not shown). The growth phenotype of this mutant in various media containing different carbon and nitrogen sources was similar to the parental strain, indicating that *AfuRbp* is not essential in *A. fumigatus*. In PCR screening of RNAi transformants, one positive integrant was selected. This transformant was grown in *glaA*-inducing medium containing 1% maltodextrin, as the sole carbon source. Semi-quantitative RT-PCR analysis using primers RPB-rt1 and RBP-rt2 showed the down-regulation of *AfuRbp* expression in the presence of maltodextrin (Fig. 1D). Phenotypic analysis of this transformant in inducing medium re-confirmed the non-essential role of *AfuRbp* in fungal growth.

### Effect of juglone and the synthetic compound on the mutant strains

To test whether the deletion or down-regulation of *AfuRbp* has any effect on drug susceptibility of the fungus, MIC levels were determined for phenylglyoxal, juglone, and JA. Table 3 demonstrates MIC values for these compounds. The results demonstrated a higher sensitivity of all strains to juglone compared to phenylglyoxal and JA. While the phenylglyoxal compound did not appear as a strong antifungal inhibitor, the mutant strains showed more sensitivity to this chemical. MIC assays for RNAi transformant was performed in maltodextrin medium. The higher sensitivity of RNAi transformant in maltodextrin medium compared to the glucose medium indicated that the down-regulation of *AfuRbp* can affect the strain susceptibility to PPI inhibitors.

**Table 3:** *In vitro* activity of compounds against wild-type and *AfuRbp* mutants of *A. fumigatus* by MIC test (µg/ml)

Inhibitor	Wild strain		KO mutant		RNA_i_ mutant	
		
	RPMI/glucose	RPMI/maltodextrin	RPMI/glucose	RPMI/maltodextrin	RPMI/glucose	RPMI/maltodextrin
Juglone	3.12	3.12	1.56	1.56	3.12	1.56
Phenylglyoxal	200	200	100	100	200	100
Juglone acetate	6.25	6.25	3.12	3.12	6.25	3.12
Amphotericin B	1.56	1.56	1.56	1.56	1.56	1.56

### Outcome of modeling and docking for AfuRBP as a model

**Fig. 3:**
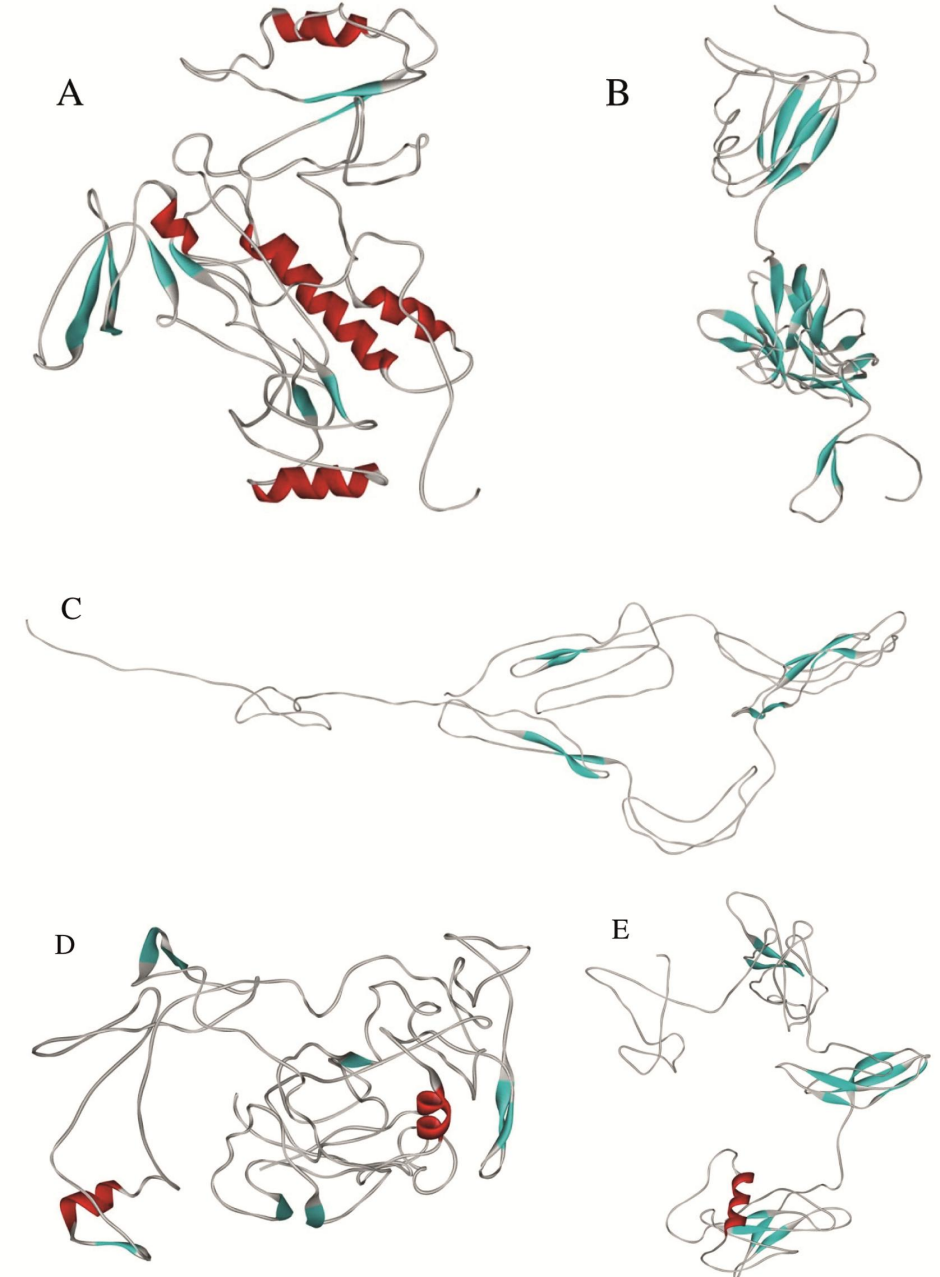
The five predicted three-dimensional structures of AfuRBP protein obtained by homology modeling with I-TASSER. Model numbers: 1 (A), 2 (B), 3 (C), 4 (D), 5 (E).

As we could not find any template protein having a minimum 35% similarity to our target protein, a threading algorithm for RBP protein modeling was used by I-TASSER server. Five models were generated, and according to the I-TASSER validation criteria, model number one had the least calculation errors and fitted best within the X-ray/NMR 3D population criteria (Fig. 3). In this study, five top models proposed by I-TASSER were used as receptors of the docking experiments, and phenylglyoxal, juglone, and JA were used as ligands. Docking outcome showed that the interaction of model number 4 with JA produced a proper energy (-186.3), which indicates the highest absolute value among the docking results. Other docking outcomes that were sorted by energy are shown in Table 4.

**Table 4:** The results of Docking experiments

Dock name	Produced energy of interaction (kJ/mol)	Position (supercoil)	Position (alphaa helix)	Position (beta sheet)
Model4 with JA	-186.3	8-9-10-11-243-244-275-317	245-246-247	276-277
Model3 with JA	-184.7	347-348-349-350-366-367-368-369-370-371	None	317-318-319-320-321
Model1 with JA	-174.5	220-221-222-223-224-225-226-227-228-229-230-231-232-233-234-235-236	237-238-239-240	None
Model5 with JA	-174.4	109-110-111-112-113-114-115-131-132-133-134-135-136-181-182-183	None	None
Model3 with juglone	-166.4	324-325-350-356-357-358-363-364	None	None
Model2 with JA	-162.5	80-81-82-83-84-85-126-127-128	None	79-86-87
Model4 with juglone	-161.7	74-75-156-157-158-351-352	None	None
Model3 with phenylglyoxal	-153.12	354-355-356	None	None
Model1 with juglone	-152	26-27-28-29-30-31-213-214-215-216	None	None
Model4 with phenylglyoxal	-146.5	383-384-385	None	None
Model1 with phenylglyoxal	-146.1	243-244-245-246-247	279-280	None
Model2 with phenylglyoxal	-143.5	143-144-145-157-158	229-230-231	219-220-221
Model5 with phenylglyoxal	-117.3	167-168-169-170-171-172-173-174-175	None	None
Model5 with juglone	-31.9	82-83-84-85-86-87	None	126-127
Model2 with juglone	0	None	None	None

The synthetic compound (JA), juglone and phenylglyoxal, were used as ligands and the five predicted models (1, 2, 3, 4, and 5) were used as receptors. Position of docked ligand and binding sites for docking results with their energy values are indicated in the Table. Docking was performed by HEX 6.3 software.

## DISCUSSION

Because of the common eukaryotic origin of fungi and mammals, the development of new antifungal drugs has been challenging. This may be the reason for the introduction of only five classes of antifungal agents, including fluorinated pyrimidine analogs, polyenes, allylamines, azoles, and echinocandins[22]. Here, we have tried to find some novel drug targets against *A. fumigatus* using bioinformatics. Our alignment has demonstrated a group of proteins/genes (Table 2) that are fungal-specific and may be essential to *A. fumigatus*. Our highlights differ from other studies such as Abadio and colleagues[23] in comparative alignment as we searched for those novel targets that only exist in *A. fumigatus*. In this study, we found a RNA-binding protein (AfuRBP) that has been annotated as a member of PPI enzyme family. The PPI family contains three members, including cyclophilins, FKBPs, and Parvulin[24]. Cyclophilins are described by an eight-standard β-barrel that forms a hydrophobic pocket. FKBPs, instead, contain an amphipatic, five-stranded β-sheet that enfolds around a single, short α-helix[25, 26]. Members of Parvulin family contain a PPI domain consisting of a half β-barrel and its four antiparallel strands surrounded by four α-helices. The results of I-TASSER modeling and docking demonstrated that the AfuRBP is structurally closer to Parvulin family members of PPI enzyme family. To elucidate the function of this protein, *AfuRbp*-deficient strains were generated using gene deletion and RNAi strategies. Normal phenotype of these strains indicated a non-essential role for this protein in growth physiology of *Aspergillus fumigatus*. Some studies have demonstrated that the inactivation of a drug target gene can result in increased sensitivity of the organism to the drug or other compounds that inhibit the same pathway[27, 28]. In this sense, the sensitivity of *AfuRbp*-disrupted strains to PPI inhibitors was assessed.

Juglone showed the highest inhibitory effect on the mutants and wild strain. In addition, we used JA as an inhibitor to investigate the change in the structure of juglone through modification of its hydroxyl group in order to increase the hydrophobicity. The decision to produce JA was partly due to the predicted activity of this derivative through the docking scores, which had a higher absolute energy of interaction than juglone. Overall, MIC test of JA demonstrated different results for the mutant and wild strains. Although docking showed a high potential for JA, the MIC test indicated slight decrease in activity, which might be due to lipophilic binding of JA to other cell components.

Considering the specific inhibitory effect of juglone on Parvulin[29] and the higher sensitivity of *AfuRbp* deficient mutants to this chemical compared to the parental strain, it can be concluded that juglone may act as a specific inhibitor of the AfuRBP. More detailed studies on recombinant AfuRBP may verify this claim.

In conclusion, in this study, the bioinformatics and biological assessments of AfuRBP were carried out, and the presented data can be considered as a start for pin pointing a target towards developing a new drug against *A. fumigatus*.

## References

[1] Nicolle LE, Rotstein C, Bourgault AM, St-Germain G, Garber G (1998). Registry CIDSIF Or the Canadian Infectious Diseases Society Invasive Fungal Registry. Invasive fungal infections in Canada from 1992 to 1994. The canadian journal of infectious diseases.

[2] Nivoix Y, Velten M, Letscher-Bru V, Moghaddam A, Natarajan-Amé S, Fohrer C, Lioure B, Bilger K, Lutun P, Marcellin L, Launoy A, Freys G, Bergerat JP, Herbrecht R (2008). Factors associated with overall and attributable mortality in invasive Aspergillosis. Clinical infectious diseases.

[3] Oliver JD, Sibley GE, Beckmann N, Dobb KS, Slater MJ, McEntee L, du Pre S, Livermore J, Bromley MJ, Wiederhold NP, Hope WW, Kennedy AJ, Law D, Birch M (2016). F901318 represents a novel class of antifungal drug that inhibits dihydroorotate dehydrogenase. Proceedings of the national academy of sciences of the United States of America.

[4] Lavecchia A, Di Giovanni C (2013). Virtual screening strategies in drug discovery: a critical review. Current medicinal chemistry.

[5] Barh D, Kumar A (2009). In silico identification of candidate drug and vaccine targets from various pathways in Neisseria gonorrhoeae. In silico biology.

[6] Carlton JM, Angiuoli SV, Suh BB, Kooij TW, Pertea M, Silva JC, Ermolaeva MD, Allen JE, Selengut JD, Koo HL, Peterson JD, Pop M, Kosack DS, Shumway MF, Bidwell SL, Shallom SJ, van Aken SE, Riedmuller SB, Feldblyum TV, Cho JK, Quackenbush J, Sedegah M, Shoaibi A, Cummings LM, Florens L, Yates JR, Raine JD, Sinden RE, Harris MA, Cunningham DA, Preiser PR, Bergman LW, Vaidya AB, van Lin LH, Janse CJ, Waters AP, Smith HO, White OR, Salzberg SL, Venter JC, Fraser CM, Hoffman SL, Gardner MJ, Carucci DJ (2002). Genome sequence and comparative analysis of the model rodent malaria parasite Plasmodium yoelii yoelii. Nature.

[7] Butt AM, Nasrullah I, Tahir S, Tong Y (2012). Comparative genomics analysis of *Mycobacterium ulcerans* for the identification of putative essential genes and therapeutic candidates. PloS one.

[8] Nierman WC, Pain A, Anderson MJ, Wortman JR, Kim HS, Arroyo J, Berriman M, Abe K, Archer DB, Bermejo C, Bennett J, Bowyer P, Chen D, Collins M, Coulsen R, Davies R, Dyer PS, Farman M, Fedorova N, Fedorova N, Feldblyum TV, Fischer R, Fosker N, Fraser A, García JL, García MJ, Goble A, Goldman GH, Gomi K, Griffith-Jones S, Gwilliam R, Haas B, Haas H, Harris D, Horiuchi H, Huang J, Humphray S, Jiménez J, Keller N, Khouri H, Kitamoto K, Kobayashi T, Konzack S, Kulkarni R, Kumagai T, Lafon A, Latgé JP, Li W, Lord A, Lu C, Majoros WH, May GS, Miller BL, Mohamoud Y, Molina M, Monod M, Mouyna I, Mulligan S, Murphy L, O'Neil S, Paulsen I, Peñalva MA, Pertea M, Price C, Pritchard BL, Quail MA, Rabbinowitsch E, Rawlins N, Rajandream MA, Reichard U, Renauld H, Robson GD, Rodriguez de Córdoba S, Rodríguez-Peña JM, Ronning CM, Rutter S, Salzberg SL, Sanchez M, Sánchez-Ferrero JC, Saunders D, Seeger K, Squares R, Squares S, Takeuchi M, Tekaia F, Turner G, Vazquez de Aldana CR, Weidman J, White O, Woodward J, Yu JH, Fraser C, Galagan JE, Asai K, Machida M, Hall N, Barrell B, Denning DW (2005). Genomic sequence of the pathogenic and allergenic filamentous fungus Aspergillus fumigatus. Nature.

[9] Kiełbasa SM, Wan R, Sato K, Horton P, Frith MC (2011). Adaptive seeds tame genomic sequence comparison. Genome research.

[10] Abouelhoda MI, Stefan Kurtz, Enno Ohlebusch (2004). Replacing suffix trees with enhanced suffix arrays. Journal of discrete algorithms.

[11] Kurtz S, Phillippy A, Delcher AL, Smoot M, Shumway M, Antonescu C, Salzberg SL (2004). Versatile and open software for comparing large genomes. Genome biol.

[12] Johnson M, Zaretskaya I, Raytselis Y, Merezhuk Y, McGinnis S, Madden TL (2008). NCBI BLAST: a better web interface. Nucleic acids research.

[13] Roy A, Kucukural A, Zhang Y (2010). I-TASSER: a unified platform for automated protein structure and function prediction. Nature protocols.

[14] Roy A, Dong Xu, Jonathan Poisson, Yang Zhang (2011). A protocol for computer-based protein structure and function prediction. Journal of visualized experiments.

[15] Zhang Y (2008). I-TASSER server for protein 3D structure prediction. BMC bioinformatics.

[16] Green MR, Sambrook J (2014). Molecular cloning a laboratory manual.

[17] Vogel H (1956). A convenient growth medium for Neurospora (medium N). Microbial genetics bulletin.

[18] Khalaj V, Azizi M, Enayati S, Khorasanizadeh D, Ardakani EM (2012). NCE102 homologue in Aspergillus fumigatus is required for normal sporulation, not hyphal growth or pathogenesis. FEMS microbiology letters.

[19] Khalaj V, Eslami H, Azizi M, Rovira-Graells N, Bromley M (2007). Efficient downregulation of alb1 gene using an AMA1-based episomal expression of RNAi construct in Aspergillus fumigatus. FEMS microbiology letters.

[20] Bromley M, Gordon C, Rovira-Graells N, Oliver J (2006). The Aspergillus fumigatus cellobiohydrolase B (cbhB) promoter is tightly regulated and can be exploited for controlled protein expression and RNAi. FEMS microbiology letters.

[21] van Hartingsveldt W, Mattern IE, van Zeijl CM, Pouwels PH, van den Hondel CA (1987). Development of a homologous transformation system for Aspergillus niger based on the pyrG gene. Molecular genetics and genomics.

[22] Cannon RD, Lamping E, Holmes AR, Niimi K, Baret PV, Keniya MV, Tanabe K, Niimi M, Goffeau A, Monk BC (2009). Efflux-mediated antifungal drug resistance. Clinical microbiology reviews.

[23] Abadio AK, Kioshima ES, Teixeira MM, Martins NF, Maigret B, Felipe MS (2011). Comparative genomics allowed the identification of drug targets against human fungal pathogens. BMC genomics.

[24] Shaw PE (2002). Peptidyl-prolyl isomerases: a new twist to transcription. EMBO reports.

[25] Michnick SW, Rosen MK, Wandless TJ, Karplus M, Schreiber SL (1991). Solution structure of FKBP, a rotamase enzyme and receptor for FK506 and rapamycin. Science.

[26] Van Duyne GD, Standaert RF, Karplus PA, Schreiber SL, Clardy J (1991). Atomic structure of FKBP-FK506, an immunophilin-immunosuppressant complex. Science.

[27] Giaever G, Shoemaker DD, Jones TW, Liang H, Winzeler EA, Astromoff A, Davis RW (1999). Genomic profiling of drug sensitivities via induced haploinsufficiency. Nature genetics.

[28] Alam MK, El-Ganiny AM, Afroz S, Sanders DA, Liu J, Kaminskyj SG (2012). Aspergillus nidulans galactofuranose biosynthesis affects antifungal drug sensitivity. Fungal genetics and biology.

[29] Hennig L, Christner C, Kipping M, Schelbert B, Rücknagel KP, Grabley S, Küllertz G, Fischer G (1998). Selective inactivation of parvulin-like peptidyl-prolyl cis/trans isomerases by juglone. Biochemistry.

